# P-657. *Mycoplasma pneumoniae* Pneumonia in Korean Children and Adolescents in 2023, Multicenter Study

**DOI:** 10.1093/ofid/ofae631.854

**Published:** 2025-01-29

**Authors:** Ki Wook Yun, Hye Jeong Moon, Joon Kee Lee, Taekjin Lee, Youngmin Cho, Da Yun Kang, Hyunju Lee, Eun Hwa Choi

**Affiliations:** Seoul National University Children's Hospital, Seoul, Seoul-t'ukpyolsi, Republic of Korea; Seoul National University Children's Hospital, Seoul, Seoul-t'ukpyolsi, Republic of Korea; Chiungbuk National Hospital, Cheongju, Ch'ungch'ong-namdo, Republic of Korea; CHA Bundang Medical Center, Seongnam, Kyonggi-do, Republic of Korea; Seoul National University Bundang Hospital, Seongnam-si, Kyonggi-do, Republic of Korea; Department of Pediatrics, Seoul National University Children’s Hospital, Seoul, South Korea, seoul, Seoul-t'ukpyolsi, Republic of Korea; Seoul National University Bundang Hospital, Seongnam-si, Kyonggi-do, Republic of Korea; Seoul National University Children's Hospital, Seoul, Seoul-t'ukpyolsi, Republic of Korea

## Abstract

**Background:**

*Mycoplasma pneumoniae* (MP) is the leading cause of community-acquired pneumonia in the post-pneumococcal conjugate vaccine era, particularly in children. Following the COVID-19 pandemic, a resurgence of MP pneumonia has been observed. Thus, we aimed to investigate the clinical manifestations, macrolide resistance patterns, and therapeutic approaches related to the recent MP pneumonia epidemic.

Numbers and percentage of antibiotics and corticosteroid utilization in managing Mycoplasma pneumoniae pneumonia

**Figure:**
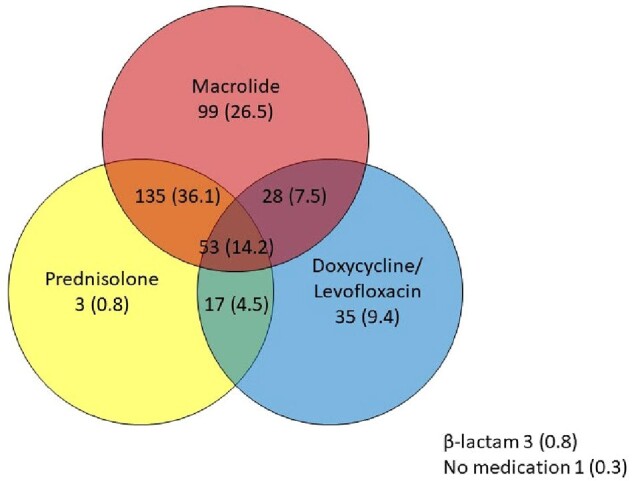

**Methods:**

This multicenter study, conducted in South Korea, enrolled children and adolescents diagnosed with MP pneumonia between September and December 2023, spanning outpatient clinics and hospitalized settings. Clinical data were retrospectively collected from 13 referring hospitals using standardized microbiological criteria, including a positive PCR result or a four-fold increase in serologic tests. Statistical analyses were then performed to evaluate demographic characteristics, treatment modalities, and clinical outcomes.

Treatment options for Mycoplasma pneumoniae pneumonia according to defervescence

**Figure:**
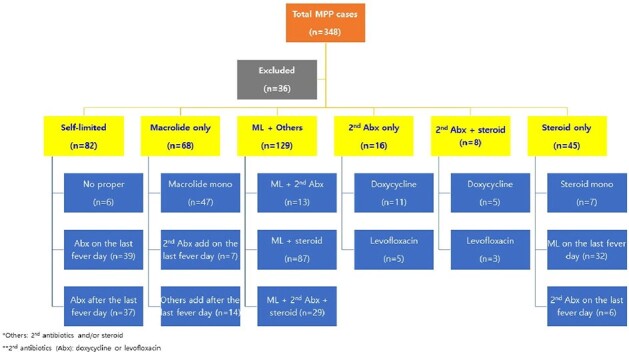

**Results:**

In total, 474 patients were enrolled, of whom 374 met the microbiological confirmation criteria. The median age of the patients was 7.7 (IQR, 5.4–9.6) years, and the hospitalization rate was 88.6%. Most patients experienced fever (98.9%), and lobular/lobar consolidation (59.1%) was the most common radiological finding. The macrolide resistance rate remained high at 87.0% but did not significantly influence the radiological findings or outcomes. Patients with consolidation showed a longer fever duration (median 8 vs. 7 days, P = 0.020) and higher hospitalization rates (92.3% vs. 83.0%, P = 0.008). Macrolides and steroids were used in 83.0% and 50.6%, respectively. A total of 82 (23.6%) participants were considered to have defervesced naturally in 5 (IQR 4-7) days. The total duration of fever was 7 (IQR 5-9) days in those who used only macrolides (n=68, 19.5%) and 8 (IQR 6-10) days in those who used macrolides with other treatments (tetracyclines, quinolones, or steroids; n=129, 37.1%). There was no difference in complications or readmission rates between the two groups.

Therapeutic Management and Clinical Response of Children and Adolescents with Mycoplasma pneumoniae Pneumonia

**Figure:**
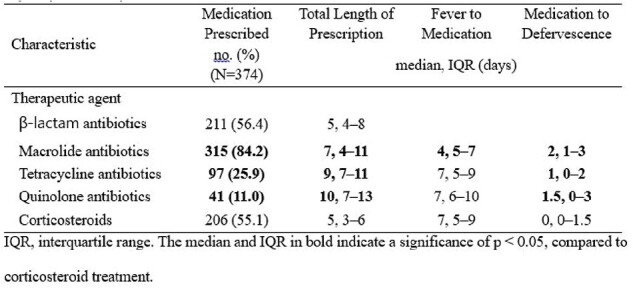

**Conclusion:**

This study highlights the ongoing challenge of macrolide resistance in MP pneumonia and the need for tailored therapeutic approaches. Macrolides remain commonly prescribed despite high resistance; corticosteroids are often used concurrently.

Comparison of Clinical Characteristics, Management, and Outcome of Mycoplasma pneumoniae Infection According to Radiologic Findings

**Figure:**
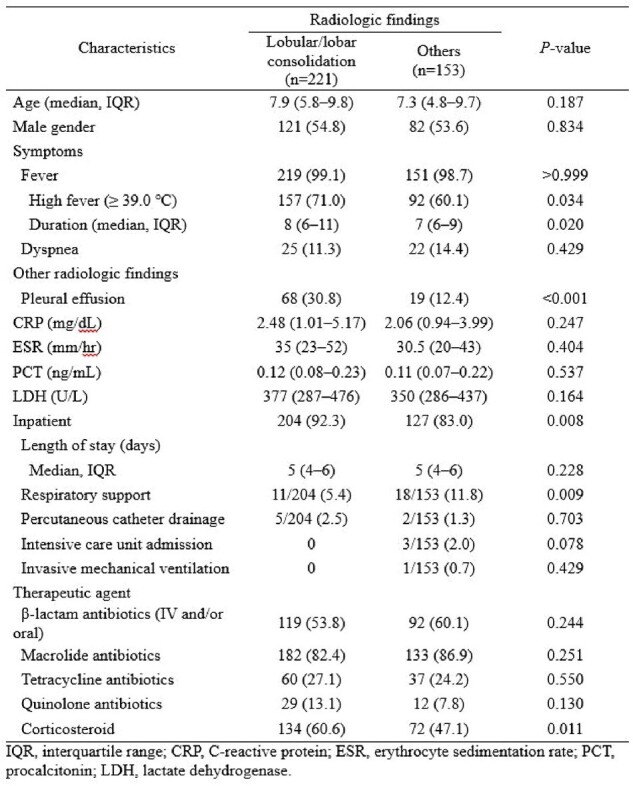

**Disclosures:**

**All Authors**: No reported disclosures

